# Characteristics and Outcomes of Patients Receiving Sedation for Voiding Cystourethrography

**DOI:** 10.7759/cureus.20207

**Published:** 2021-12-06

**Authors:** Keith A Hanson, Shane C Rainey, Nadia Shaikh, Michele K Beekman

**Affiliations:** 1 Pediatrics, University of Illinois College of Medicine at Peoria, Peoria, USA; 2 Child Health, University of Arizona College of Medicine - Phoenix, Phoenix, USA

**Keywords:** voiding cystourethrography, propofol, urology, radiology, procedural sedation

## Abstract

Background

Voiding cystourethrography (VCUG) is used to diagnose vesicoureteral reflux (VUR); however, it is an invasive procedure and can be psychologically distressing. Procedural sedation is occasionally utilized to alleviate anxiety during VCUG, and some patient populations may get referred more readily for sedation than others. Sedative medications may also impact the results of the test due to their effects on smooth muscle. The goals of this study were to compare patient characteristics between those that were referred for procedural sedation and those that were not and to compare VCUG results between sedated and non-sedated patients.

Methodology

We performed a retrospective cohort study of patients aged 2-18 years undergoing VCUG during a five-year period. Sedated patients were matched with non-sedated patients controlling for referring provider and procedure year. Exclusion criteria included chronic catheterization, same-day surgery, current intensive care admission, and sedation restrictions. A total of 284 patients were included. Demographic information, medical comorbidities, and VCUG results were analyzed.

Results

There were no significant differences between sedated and non-sedated patients in any demographic variables. Neurologic, developmental, and gastrointestinal comorbidities were more common in sedated patients. On multivariate analysis, having more than one comorbid condition was the only significant predictor of referral for procedural sedation. There were no significant differences in VCUG results between sedated and non-sedated patients.

Conclusions

Patients with comorbidities were more likely to receive procedural sedation for VCUG. Procedural sedation did not have a significant impact on test results, suggesting its potential utility in relieving pain and anxiety associated with VCUG.

## Introduction

Vesicoureteral reflux (VUR) is a relatively common pediatric diagnosis with an uncertain natural course, although it can lead to recurrent urinary tract infections (UTI) and subsequent renal scarring. Voiding cystourethrography (VCUG) remains the gold standard in the diagnosis of VUR and must be performed accurately to prevent under-recognition of high-grade VUR and the potential of resulting kidney damage [1]. Unfortunately, VCUG is an invasive medical procedure and can be a painful and distressing psychological experience for patients and parents for various reasons [2]. Placing a urinary catheter is an uncomfortable event and creates an opportunity for urethral trauma in an uncooperative child, leading to possible dysuria and difficulty voiding during the procedure [3]. Furthermore, children are asked to void in unnatural environments amid unfamiliar individuals, creating further stress, anxiety, and apprehension which may predispose them to avoidable healthcare behaviors in adulthood [4,5].

To alleviate these concerns, procedural sedation has been utilized in this patient population. Unfortunately, there is no current consensus on which children should undergo sedation for VCUG and selection varies widely depending on clinician preference. Additionally, employing deep sedation during VCUGs remains controversial as smooth muscle relaxing sedative medications may negatively impact voiding dynamics by limiting the full contraction of the bladder and the surrounding pelvic musculature. Studies have suggested that the voiding phase of VCUG is important for accurate detection of reflux, with one study observing that up to 42% of patients demonstrated reflux during voiding alone [6,7]. Therefore, concerns exist regarding a patient’s ability to completely void under sedation, which, in turn, may lead to inaccurate results [8]. A majority of the debate in this area concerns propofol, a common sedative with muscle relaxant properties. Relatively few studies have focused on procedural sedation during VCUG, all of which examined the use of midazolam or nitrous oxide [9-11]. In one review, midazolam was found to be effective for procedural sedation during VCUG and was not found to affect the detection or grading of VUR [12]. However, to our knowledge, no studies have addressed the use of propofol during VCUG and its subsequent effects on the detection and grading of VUR.

The objective of our study was to compare and contrast the characteristics of children who have undergone sedated versus non-sedated VCUG and to determine retrospectively if propofol sedation impairs the ability of VCUG to detect VUR. We hypothesized that medical comorbidities would be predictive of patients receiving sedation and that there would be no difference in the results of VCUG between patients who underwent procedural sedation with propofol compared to those who remained awake during the procedure.

## Materials and methods

This retrospective cohort study was conducted at a single-center, 144-bed academic tertiary care children’s hospital located within a larger healthcare system in the Midwestern United States. Approval was granted by the Peoria Institutional Review Board (project approval number: 705102). Our institution has a robust pediatric sedation service that accepts sedation referrals for VCUG based on provider preference.

The study was originally powered to detect significant differences in the likelihood of sedation referral based on demographic variables and comorbidities using a multivariable linear regression model. Using the number of planned predictor variables and a squared population multiple correlation coefficient (r^2^) of 0.4, we estimated a sample size of 170 for a good prediction level. Therefore, we planned to review approximately 200 charts.

Patients between two and eighteen years of age undergoing their first VCUG during the five-year period from March 2010 to January 2015 were eligible for inclusion into the study. Exclusion criteria included indwelling or chronic catheterization, same-day surgery, current intensive care admission, and inability to medically qualify for procedural sedation. Given that the average outpatient pediatrician may have little to no experience with the VCUG procedure, and radiology rotations are not required during pediatric residency, we expected that a group of providers familiar with the VCUG procedure (urologists) would comprise the largest portion of the referral base and may skew the results if they had a clear preference for sedation. Therefore, to control for the variable of referring provider, we categorized providers into five categories: general pediatrics (pediatrics, combined medicine-pediatrics, and family medicine), inpatient providers (hospitalists and residents on inpatient services), urologists, other specialists (nephrologists, gastroenterologists, emergency medicine, and surgeons), and advanced practice providers (advanced practice nurses and physician assistants). All sedated cases in the study duration were included in the sample, and each sedated patient was matched with a non-sedated control from the same provider category and study year. This is an important control as referral patterns may change from year to year and referrals to sedation can be highly dependent on the referring provider’s knowledge of, and experience with, the sedation service.

In our facility, VCUG is performed primarily by our fellowship-trained pediatric radiologists, assisted by radiology techs, our sedation nurses, and child life specialists. During this study, we utilized both oral midazolam (mild-to-moderate sedation) and intravenous propofol (deep sedation) to provide sedation. Patients scheduled to receive propofol were pre-screened for sedation exclusion criteria via telephone before their scheduled procedure day. When utilizing oral midazolam, the medication is typically given in the pre-operative area prior to transporting the patient to the radiology suite. The patient is then brought to the radiology suite, prepped, placed on the table, and connected to cardiac, respiratory, blood pressure, and pulse oximetry monitors. Propofol is then given by the sedation physician until deep sedation is achieved. The genitourinary area is then sterilized in the usual fashion and catheterization is performed, followed by contrast administration. The radiologist then obtains the initial images until the voiding phase, at which time propofol is discontinued to allow the patient to emerge to the point of spontaneous voiding. The catheter is then removed and the patient is taken to recovery.

The study cohort then underwent a full chart review by the study’s principal investigator. Data collected for each patient in the cohort included patient and family characteristics, provider characteristics, medical comorbidities, and sedation-related characteristics. Patient and family characteristics included age, gender, ethnicity, medical history, and insurance type. Provider groups were classified into five groups, as noted above. Pertinent medical history and diagnoses were collected from the patient’s chart during review and were classified into various categories, including neurological (any seizure disorder, cerebral palsy, hypoxic-ischemic encephalopathy), developmental (developmental delay, autism spectrum disorder, sensory integration disorder, trisomy 21, other chromosomal disorders), psychiatric (attention-deficit hyperactivity disorder, oppositional defiant disorder), cardiac (heart murmurs, atrial septal defects, ventricular septal defects, patent foramen ovale), pulmonary (any classification of asthma), gastrointestinal (constipation, functional abdominal pain, chronic abdominal pain), genitourinary (VUR, enuresis, UTI, recurrent UTI, dysfunctional voiding), and others (a family history of VUR, orthopedic conditions, allergic disorders, autoimmune disorders, hematologic or oncologic conditions). It was also noted whether the patient had a previous VCUG prior to the study period (the index study was the first performed during the years listed). VCUG data included whether the study was normal or abnormal, and the grade of VUR was identified on the standard 1-5 scale [13]. Procedural sedation characteristics included the specific medications utilized, their route of administration, and any complications related to sedation. We defined procedural sedation consistent with the 2002 practice guidelines from the American Society of Anesthesiologists [14]. Patient charts were also evaluated to determine if they had a follow-up VCUG, and if so, data were collected on those patients and included the use of procedural sedation and the degree of VUR identified during the subsequent study.

Statistical analysis

Descriptive statistics were calculated overall as well as in each cohort. For continuous data, mean and standard deviation describe normally distributed data. P-values were obtained by utilizing the following tests: chi-square ​​and McNemar’s test for categorical data, paired t-test for continuous and normally distributed data (age), and Wilcoxon rank tests for skewed continuous data (VUR grade). Multivariate logistic regression models were used to determine if there were patient or procedural characteristics that were predictive of being referred for procedural sedation, as well as whether receiving sedation was predictive of an abnormal test result.

## Results

**Figure 1:**
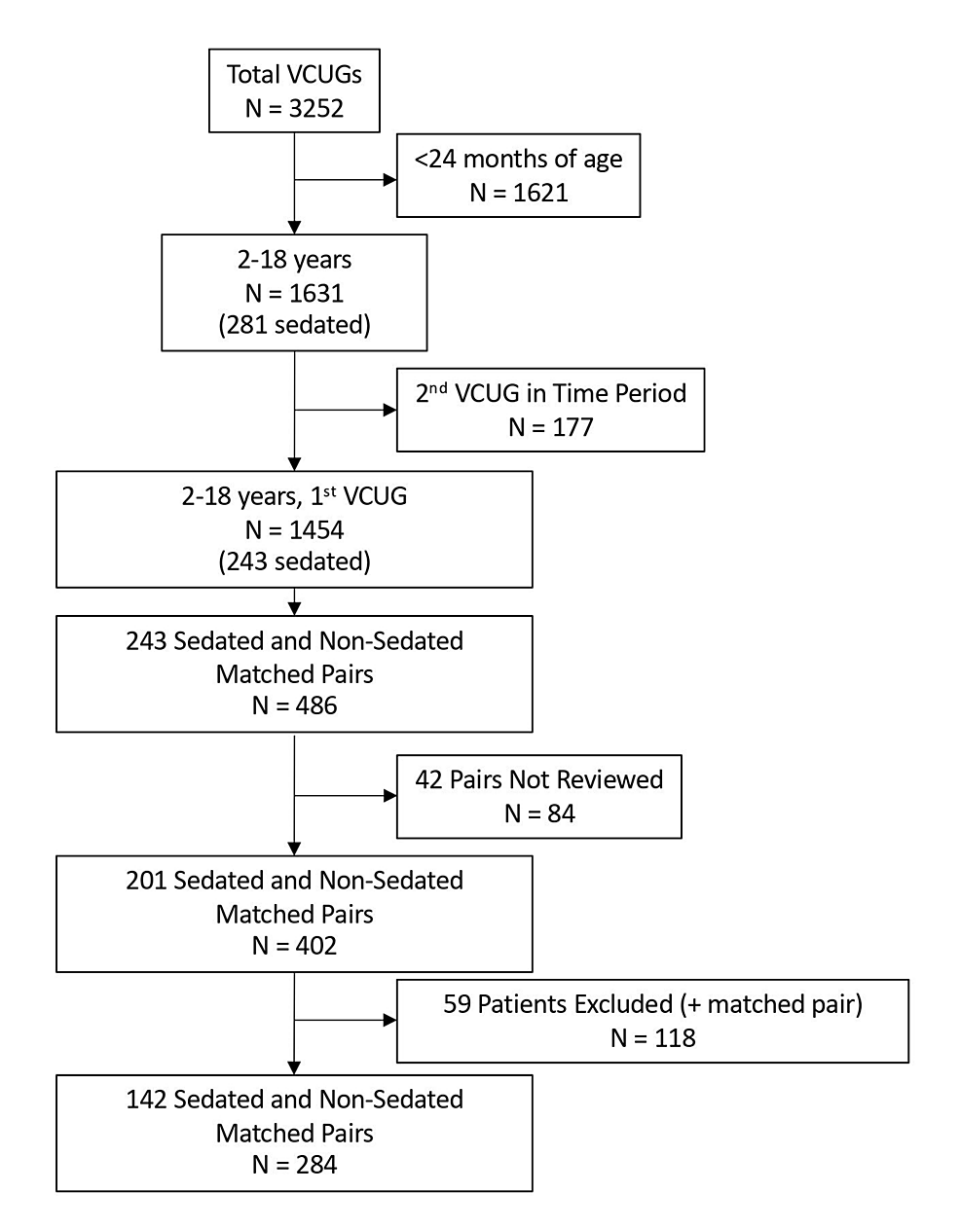
Flowchart of patients included in the study. VCUG: voiding cystourethrogram

Over the five-year study period, 3,252 VCUGs were performed at our institution. Of these, 1,631 were performed on patients between two and eighteen years of age, with 281 sedated and 1,350 non-sedated studies. Overall, 1,454 patients underwent their first VCUG, 243 sedated and 1,211 non-sedated, and thus met our inclusion criteria. The 243 sedated patients were matched with a random control patient for the study year and provider group for a total of 486 matched encounters. Overall, 84 charts (42 pairs) were not reviewed due to having exceeded the goal number of charts based on our power calculation. A total of 59 sedated patients and their controls (118 patients) were excluded based on the exclusion criteria (Figure 1). Therefore, 284 (142 pairs) patients were included in the final patient sample. The average patient age was 5.2 years, with a median age of four years and an interquartile range of four years.

There was no significant difference on univariate analysis in any demographic variables between patients receiving procedural sedation compared with those that did not (Table 1). Several medical comorbidities were significantly more likely to get a referral for procedural sedation, including neurological, developmental, gastrointestinal, and others (Table 2). Patients with other significant medical comorbidities included a family medical history of VUR, orthopedic diagnoses, allergic disorders, immune disorders, and hematologic and oncologic diseases. On multivariate logistic regression, having more than one medical comorbidity was the only significant predictor of sedation referral (odds ratio [OR] = 3.48, p < 0.001) (Table 3).

**Table 1:** Demographic comparison between sedated and non-sedated patients. VCUG: voiding cystourethrogram; SD: standard deviation

	No sedation (n = 142)	Sedation (n = 142)	P-value
Age, mean (SD)	5.21 (3.62)	5.15 (2.95)	0.874
Female gender, n (%)	124 (87.3)	114 (80.3)	0.105
Caucasian ethnicity, n (%)	125 (88)	121 (85.2)	0.479
Private insurance, n (%)	72 (50.7)	78 (54.9)	0.493
No previous VCUG, n (%)	103 (72.5)	100 (70.4)	0.680

**Table 2:** Comparison of medical comorbidities between sedated and non-sedated patients.

	No sedation (n = 142)	Sedation (n = 142)	P-value
Neurological, n (%)	1 (0.7)	9 (6.3)	0.011
Developmental, n (%)	3 (2.1)	20 (14.1)	<0.001
Psychiatric, n (%)	12 (8.5)	16 (11.3)	0.41
Cardiac, n (%)	4 (2.8)	3 (2.1)	0.71
Pulmonary, n (%)	8 (5.6)	16 (11.3)	0.07
Endocrine, n (%)	2 (1.4)	2 (1.4)	1.00
Gastrointestinal, n (%)	28 (19.7)	47 (33.1)	0.009
Genitourinary/Renal, n (%)	137 (96.5)	139 (97.9)	0.48
Others, n (%)	17 (12)	36 (25.4)	0.004

**Table 3:** Multivariate logistic regression for the likelihood of sedation referral based on patient demographics. Only having more than one medical comorbidity was associated with sedation referral. VCUG: voiding cystourethrogram

	P-value	Odds ratio	95% Confidence interval	
Comorbidity (ref: ≤1)	<0.001	3.48	1.84	6.56
Age in years	0.54	0.98	0.90	1.06
Gender (ref: female)	0.08	1.96	0.92	4.19
Payor (ref: Medicaid/uninsured)	0.57	1.17	0.68	1.99
Ethnicity (ref: Caucasian)	0.38	1.42	0.66	3.07
Previous VCUG (ref: No)	0.35	1.51	0.63	3.59

**Figure 2:**
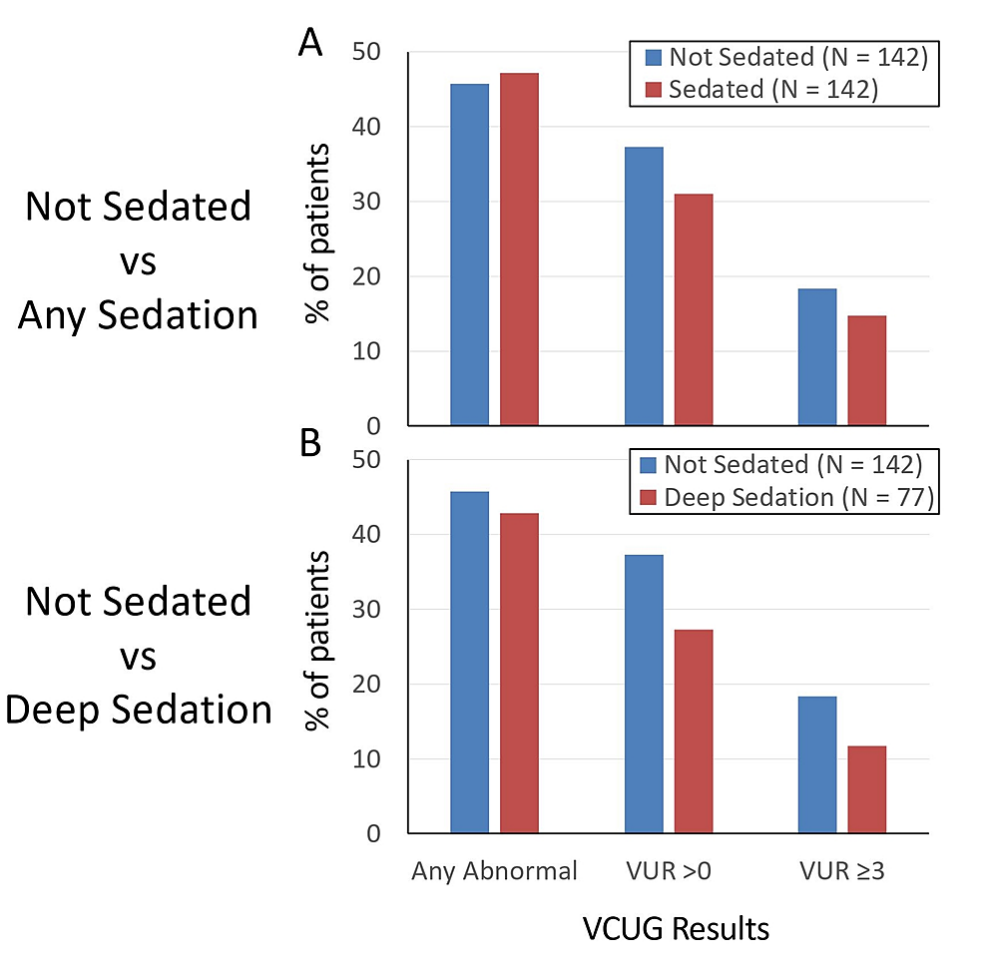
Effect of sedation on VCUG results. Panel A compares sedated VCUGs to unsedated VCUGs. Panel B compares deep sedation with propofol specifically to unsedated studies. No differences are significant (chi-square). Abnormal VCUG results in addition to VUR included anatomic abnormalities and emptying dysfunction. VCUG: voiding cystourethrogram; VUR: vesicoureteral reflux

**Figure 3:**
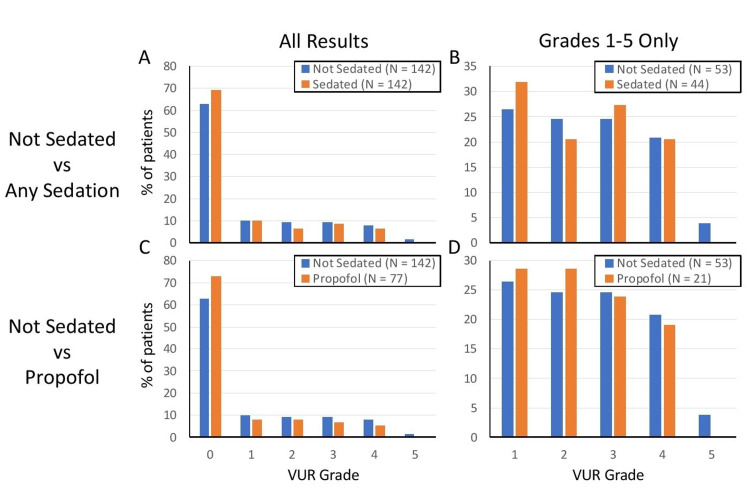
Effect of sedation on VUR grade distribution. Panels A and B compare sedated VCUGs to unsedated VCUGs, and panels C and D compare sedation with propofol specifically to unsedated studies. For comparison of VUR grades, both all results were considered (panels A and C) or only positive results (panels B and D). No difference was seen in any comparison (Wilcoxon rank sum test). VUR: vesicoureteral reflux; VCUG: voiding cystourethrogram

VCUG results were compared between sedated and unsedated patients (Figures 2A, 3A, 3B; Table 4). There were no significant differences in VCUG results between sedated and non-sedated patients; these comparisons included the percentage of patients with any abnormality, with any grade of VUR, or with significant VUR of grade 3 or higher (Figure 2; Table 4). Abnormalities seen besides VUR included bladder wall thickening, abnormal bladder shape or diverticulum, or other types of emptying dysfunction. There was also no difference in the distribution of VUR grade between groups, both when including all VUR results (grade 0-5) and when including just positive results (grade 1 or higher) (Figure 3; Table 4).

**Table 4:** Results of VCUGs for patients who were not sedated, sedated, or deeply sedated with propofol specifically. The top two rows show patients with a normal study compared to those with any abnormality. The remainder of the rows show the distribution of VUR grade for each group. No comparisons were significant (chi-square or Wilcoxon rank sum test). Abnormal VCUG results in addition to VUR include anatomic abnormalities and emptying dysfunction. VCUG: voiding cystourethrogram; VUR: vesicoureteral reflux

		Not sedated (n = 142)	Sedated (n = 142)	Deep sedation (n = 77)
Normal, n (%)		77 (54)	75 (53)	44 (57)
Abnormal, n (%)		65 (46)	67 (47)	33 (43)
VUR	0, n (%)	89 (63)	98 (69)	56 (73)
	1, n (%)	14 (10)	14 (10)	6 (8)
	2, n (%)	13 (9)	9 (6)	6 (8)
	3, n (%)	13 (9)	12 (8)	5 (6)
	4, n (%)	11 (8)	9 (6)	4 (5)
	5, n (%)	2 (1)	0 (0)	0 (0)

During the time period of the study, the majority of patients received either oral midazolam or intravenous propofol for the procedure. Midazolam would be expected to provide mild or moderate sedation and propofol deep sedation; therefore, we performed a subanalysis on patients receiving propofol to examine the effects of deep sedation specifically. We found no significant differences when comparing patients who underwent procedural sedation with propofol specifically compared to unsedated controls (Figures 2B, 3C, 3D; Table 4).

We also performed multivariate logistic regression to determine if having received sedation was predictive of VCUG results because we found that patients with comorbidities were more likely to be referred to sedation which could be a confounder. If having certain comorbidities increases the odds of having VUR, and these patients are more likely to be referred to sedation (which may affect the result), then we may find no difference where one exists. Regression was performed using age, gender, presence of more than one comorbidity, and administration of any sedation as predictors. This was done for the same outcomes shown in Figure 2: any abnormality on VCUG, any grade of VUR, or only high-grade VUR. Age and having a comorbidity were significantly associated with having any grade of VUR on multivariate analysis; sedation was not associated with having VUR or any other abnormality (Table 5). Younger age was more likely to be associated with abnormal results, any grade of VUR, and high-grade VUR. Interestingly, patients with greater than one comorbidity were significantly less likely to have any grade of VUR or high-grade VUR. The significance of this finding is not clear, especially as determining the effect of these factors on results was not the goal of the study. Finally, identical regression analyses were performed using sedation as a tiered variable, rather than binary, to delineate patients who received no sedation, moderate sedation, or deep sedation. The same pattern of significance as noted above was seen (data not shown).

**Table 5:** Results of multivariate logistic regression looking at predictors (rows) of different VCUG result outcomes (columns). Values are p-values for the coefficient to denote significance. Sedation was not a significant predictor of any outcome. Abnormal VCUG results in addition to VUR include anatomic abnormalities and emptying dysfunction. VCUG: voiding cystourethrogram; VUR: vesicoureteral reflux

	Any abnormality	Any VUR	High-grade VUR (>3)
Age	<0.001	0.001	0.022
Gender (ref: Female)	0.512	0.762	0.295
Comorbidity (ref: ≤1)	0.083	0.003	0.041
Sedation (ref: No)	0.498	0.662	0.746

For follow-up VCUGs, 25 of 237 patients with lower-grade VUR (0-2) underwent repeat studies, 14 of whom were sedated for the initial procedure (half with propofol). Of those, two patients had higher-grade reflux on repeat testing, increasing from grade 1 in both initial studies to grade 2 and grade 3 on follow-up testing. Both of these patients were sedated with propofol for the initial study. The remainder of the patients had stable or improved VUR grades.

## Discussion

VUR is a common pediatric diagnosis that relies on VCUG as its gold standard diagnostic tool. The VCUG procedure is invasive, painful, and often psychologically distressing to patients and their caregivers, which may predispose patients to avoid future contact with the healthcare system [4,5,15]. Numerous non-pharmacologic interventions are deployed to alleviate the pain and anxiety related to this procedure, including cognitive behavioral therapy, parental education and preparation, play therapy, and child life specialists, among others [3,15]. When these measures are insufficient or undesirable, procedural sedation can be utilized at the discretion of the referring clinician.

Unfortunately, there is a relative paucity of literature regarding which children will benefit from procedural sedation compared to non-pharmacologic interventions alone. Furthermore, there is little data regarding which patients are ideal candidates for referral to a procedural sedation service before undergoing VCUG [9,10]. Our study attempted to answer this question by analyzing various characteristics of patients who were referred to sedation. To our knowledge, this is the first study to compare and contrast patient-level characteristics and their effect on procedural sedation referral for VCUG.

Somewhat surprisingly, no differences were noted between sedated and non-sedated groups for age, gender, race, payor, or previous VCUG status. While not statistically significant (p = 0.08, Table 3), it was interesting that only approximately half of female patients underwent sedated VCUG compared to two-thirds of their male counterparts, given that catheterization of the female urethra is considered more technically difficult. This may be secondary to male catheter placement being more painful due to the 90-degree bend of the bulbar urethra around the external sphincter and prostate. Additionally, it was notable that the patients who had a previous VCUG before the study period were not referred for procedural sedation more frequently, given their, their family’s, and possibly their referring clinician’s previous experience with the procedure.

Our investigation was supportive of our hypothesis that children with medical comorbidities are more likely to be referred for procedural sedation for VCUG than healthy patients. On logistic regression, patients with more than one medical comorbidity were more likely to receive procedural sedation for VCUG. This is likely because these children have more prior exposure to the healthcare system and possibly invasive procedures, giving them more anxiety around healthcare encounters; they also are less likely to respond fully to non-pharmacologic distraction techniques.

Finally, we found that procedural sedation did not significantly affect VCUG results. Some providers may avoid sedation referral because of the concern that sedation medications relax smooth muscle and affect the test results; in our experience, this is frequently cited to be the case with propofol. However, using several analytical approaches, we did not see any significant difference even among patients receiving propofol. Furthermore, we did not find a substantial number of patients who were sedated for an initial test and had significantly worsened VUR on follow-up VCUG. While a relatively high proportion of patients receiving propofol (two out of seven) had higher-grade VUR on follow-up studies, the vast majority of patients with no or lower-grade reflux did not undergo follow-up studies at all, regardless of sedation status. This suggests that most of these patients do well clinically and that higher-grade disease is not widely missed because we would expect patients with higher-grade reflux to remain symptomatic and return to medical attention for further evaluation or testing. While this study was not designed or powered to determine if sedated VCUGs lead to false-negative results, and this finding is not of statistical significance, this anecdotal finding from our dataset suggests that there are not widespread spurious test results under sedation. Therefore, while our findings suggest that procedural sedation could be utilized more widely in appropriate patients without any adverse effects on study results, more studies are needed in this area.

Our study has limitations. Retrospective studies may be subject to confounders and other opportunities for bias by nature, and results must be viewed in this light. Randomizing and matching patients controlling for the referring provider and the study year was an important step in the present investigation to eliminate a potentially substantial confounder with regard to demographic and medical variables predicting sedation referral. Due to the volume of charts needing a review (1,454 patients met age, time period, and first VCUG criteria), we did not exclude patients until after matching, which gave a more reasonable number of charts to review for exclusion criteria (Figure 1). Matching and selection were randomized; however, excluding after matching could lead to selection bias. Similarly, some charts were not reviewed once our target population size was achieved based on the power calculation, which could also lead to bias, even though these were also randomly selected.

With regard to factors predicting sedation referral, while the “other” comorbidity category was found to be statistically significant, it included a family history of VUR; therefore, it may be that those families had more experience with VCUG and therefore requested sedation. Additionally, we did not match for medical comorbidities when comparing VCUG results as this was a secondary aim; thus, our study was not specifically powered to detect a difference in VCUG results, which could theoretically affect our findings. Furthermore, while our sample size was relatively large for a procedure that is not commonly done, it may be too small to provide any clinical generalizations. Future prospective studies are needed to determine the true effects propofol specifically and procedural sedation generally have on VCUG results. Moreover, other authors have shown that a percentage of grade 1 VUR can progress to grade 2-3 on subsequent studies by virtue of the disease’s natural course, without factoring in sedation [16]. Another potential confounder unaddressed in this study is variability in radiologist VCUG interpretation. While there is an international classification system for VUR, there is inherent subjectivity in the grading system [13,17]. Our study was conducted at a single institution with a single pediatric radiology group reading our studies assisted in mediating this fact. Finally, there are different anesthesia protocols and levels of sedation that may be provided by different medications, all of which may influence the ability to void and the subsequent grading of reflux.

## Conclusions

We provide novel data on patient characteristics predictive of referral for procedural sedation prior to VCUG, as well as contributing to the data regarding the effects of procedural sedation on VCUG outcome. Patients with multiple comorbidities were more likely to receive procedural sedation for VCUG. Sedation did not have a significant impact on the ability of VCUG to detect VUR, suggesting that procedural sedation is a good option to relieve anxiety and pain associated with this test. Future studies could involve similar analyses performed on a national database for wider representation and comparison.
